# Neurotoxic Antibodies against the Prion Protein Do Not Trigger Prion Replication

**DOI:** 10.1371/journal.pone.0163601

**Published:** 2016-09-29

**Authors:** Karl Frontzek, Manuela Pfammatter, Silvia Sorce, Assunta Senatore, Petra Schwarz, Rita Moos, Katrin Frauenknecht, Simone Hornemann, Adriano Aguzzi

**Affiliations:** Institute of Neuropathology, University Hospital of Zurich, University of Zurich, Zurich, Switzerland; INRA Centre de Jouy-en-Josas, FRANCE

## Abstract

Prions are the infectious agents causing transmissible spongiform encephalopathies (TSE), progressive, inexorably lethal neurological diseases. Antibodies targeting the globular domain (GD) of the cellular prion protein PrP^C^ trigger a neurotoxic syndrome morphologically and molecularly similar to prion disease. This phenomenon raises the question whether such antibodies induce infectious prions *de novo*. Here we exposed cerebellar organotypic cultured slices (COCS) to the neurotoxic antibody, POM1. We then inoculated COCS homogenates into *tg*a*20* mice, which overexpress PrP^C^ and are commonly utilized as sensitive indicators of prion infectivity. None of the mice inoculated with COCS-derived lysates developed any signs of disease, and all mice survived for at least 200 days post-inoculation. In contrast, all mice inoculated with *bona fide* prions succumbed to TSE after 55–95 days. Post-mortem analyses did not reveal any signs of prion pathology in mice inoculated with POM1-COCS lysates. Also, lysates from POM1-exposed COCS were unable to convert PrP by quaking. Hence, anti-GD antibodies do not catalyze the generation of prion infectivity. These data indicate that prion replication can be separated from prion toxicity, and suggest that anti-GD antibodies exert toxicity by acting downstream of prion replication.

## Introduction

Prion diseases are fatal neurodegenerative diseases that rely on the seeded propagation of an aggregated form of the cellular prion protein PrP^C^ [1]. The aggregated form, denoted PrP^Sc^, is typically resistant to limited digestion with proteinase K (PK). The pathology triggered by prion infections, consisting of spongiosis, neuronal loss, astrogliosis, and microglial activation, is faithfully reproduced by administration of anti-prion antibodies targeting conformational epitopes on the globular domain (GD) of PrP^C^ [2, 3]. Toxicity requires the long flexible tail (FT) of PrP^C^, and antibodies against the octapeptide repeat (OR) domain of the FT prevent the toxicity of anti-GD antibodies and antagonize neurodegeneration in prion infections [4]. Therapeutic compounds conferring anti-prion protection are frequently effective also against toxic anti-prion antibodies, suggesting that GD antibodies and *bona fide* prions share common effector pathways [4].

The striking similarities between the consequences of toxic anti-GD antibodies and of prion infections raised the question whether such antibodies might induce the generation of *de novo* prions. By distorting the conformation of PrP^C^, antibodies may conceivably catalyze the formation of higher-order aggregates that would, in turn, act as nucleation sites for the growth of PrP^Sc^ fibers [5]. This question is not only of academic importance, but it may also be of relevance to the biosafety classification of research with such antibodies. We therefore undertook to clarify whether POM1 induced *de novo* infectious prions, and if so, whether this might explain its toxicity. We treated COCS homogenates, which have similar prion propagation efficacies as whole brain homogenates [6], with the toxic anti-prion antibody POM1 and analyzed them for the presence of *de novo* prions after passaging into prion-susceptible cells and PrP^C^-overexpressing *tg*a*20* mice [7].

## Results

In order to minimize any possible effector functions and off-target effects of the antibodies, such as complement and Fcγ-binding, we generated single-chain variable fragments (scFv) of the neurotoxic anti-PrP antibody POM1 (scFvPOM1). PrP^C^-overexpressing *tg*a*20* COCS were then treated with either scFvPOM1 (400 nM) or with scFvPOM1 (400 nM) preincubated with a molar excess of recPrP_23-230_ (600 nM) for control. This treatment was maintained over 10 days with three medium changes per week; scFvPOM1 was replaced with each medium change.

**Fig 1:**
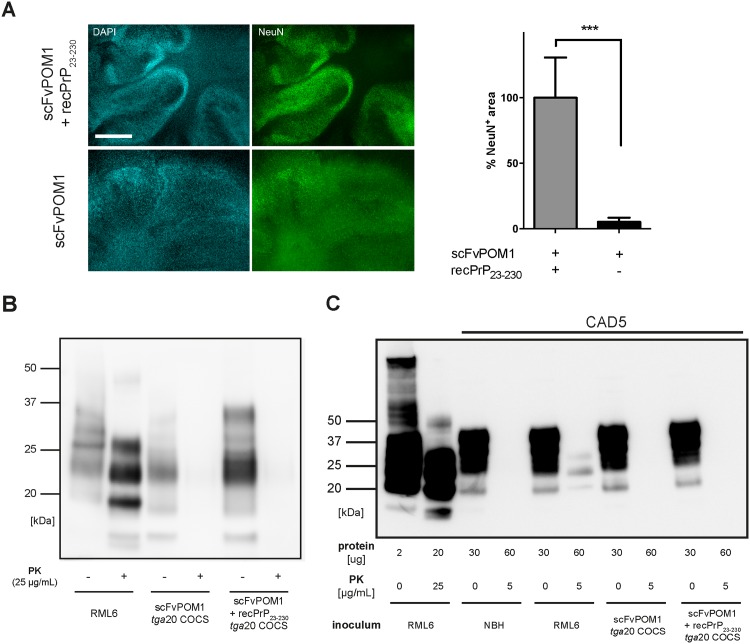
**(A)** Chronic treatment of COCS with scFvPOM1 induced profound neurodegeneration. Instead, no neurodegeneration was observed in COCS exposed to scFvPOM1 pre-incubated with recPrP_23-230_. *** p<0.001. Scale bar = 500 μm. **(B)** Pooled slice homogenates of scFvPOM1-treated (n = 8 slices) or (scFvPOM1+ recPrP_23-230_)-treated *tg*a*20* COCS (n = 5 slices) did not show PK-resistant species after digestion as is typically observed in RML6 brain homogenate (n = 1). **(C)** No PrP^Sc^ was observed after inoculation of the highly PrP^Sc^-susceptible cell line CAD5 with pooled scFvPOM1-treated COCS homogenates. CAD5 cells were successfully infected with RML6 as shown by the typical “diagnostic shift” of PK-digested RML6 with un-, mono- and diglycosylated PrP^Sc^ bands. RML6 brain homogenate served as a positive control (left band pair).

NeuN immunofluorescent stainings, which identify neurons, showed widespread neuronal degeneration in COCS treated with scFvPOM1, but not in COCS treated with antibody that had been preemptively blocked with recPrP_23-230_ (Fig 1A). To clarify whether this effect was induced by the aggregation of PrP, we analyzed pooled COCS homogenates treated with either scFvPOM1 (n = 8) or scFvPOM1 + recPrP_23-230_ (n = 5) for PrP^Sc^, an isoform of PrP that is partially resistant to proteinase K (PK) and is universally employed as a surrogate marker for prion infectivity (Figs 1B and S1) [8]. Pooled slice homogenates from scFvPOM1-treated (n = 8) and (scFvPOM1+recPrP_23-230)_)-treated (n = 5) *tg*a*20* COCS were analyzed by Western blotting and were found to be devoid of PrP^Sc^. In contrast, PK digestion of prion-containing brain homogenate (RML6 = passage 6 of the Rocky Mountain Laboratory strain mouse-adapted scrapie prions), which served as positive control, showed the typical diagnostic shift towards a smaller PK-resistant core with un-, mono- and diglycosylated PrP^Sc^.

The murine neuroblastoma cell line CAD5 is highly susceptible to prion infection and serves as a sensitive bioassay for prion propagation [9]. We hence spiked cell culture media of exponentially growing CAD5 cells with RML6 prions, non-infectious brain homogenate (NBH), or homogenates from COCS that had been exposed to scFvPOM1 or scFvPOM1+recPrP_23-230_- (6–12 ng protein of total homogenate in 1 mL of cell culture media). After 4 days of culture, we lysed the CAD5 cells and assessed PrP^Sc^ by Western blotting. No PrP^Sc^ was detectable in CAD5 cells inoculated with COCS homogenates exposed to either scFvPOM1 or scFvPOM1+recPrP_23-230_. In contrast, RML6 prion-infected CAD5 cells displayed the typical pattern of PrP^Sc^ on Western blots (Figs 1C and S2).

**Fig 2:**
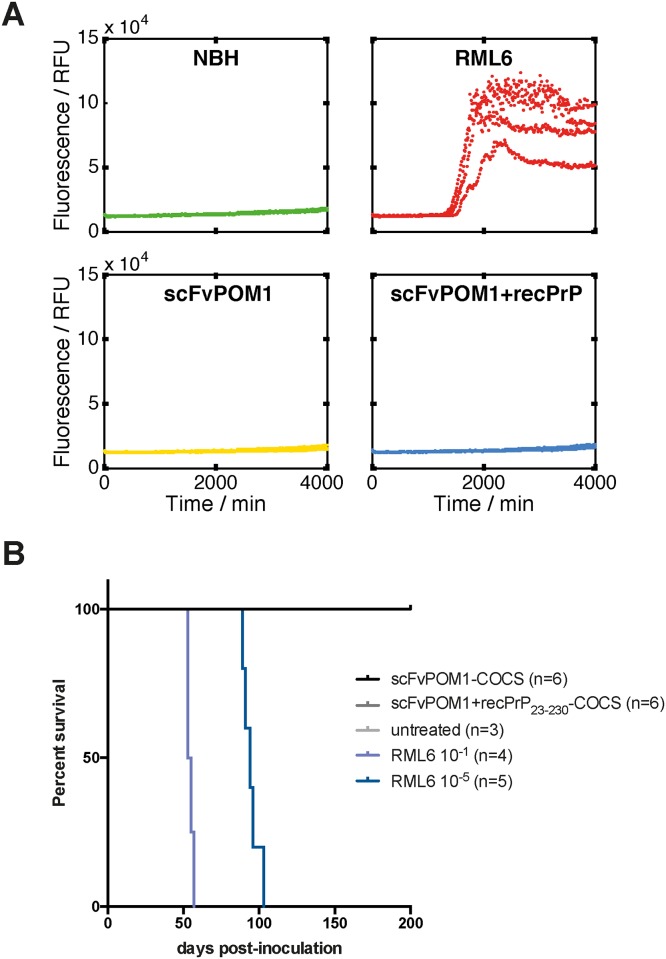
**(A)** RT-QuIC analyses failed to reveal *de novo* PrP^Sc^ aggregation when seeded with scFvPOM1 or (scFvPOM1+recPrP_23-230_)-treated *tg*a*20* COCS homogenates. NBH- and RML6-treated *tg*a*20* COCS homogenates were used as negative and positive controls for prion-catalyzed amplification of aggregated PrP, respectively. RT-QuIC reactions were performed in quadruplicates for each sample. **(B)** *tg*a*20* mice inoculated with pooled slice homogenates of scFvPOM1-treated (n = 6 mice) or (scFvPOM1+recPrP_23-230_)-treated *tg*a*20* COCS (n = 6 mice) stayed healthy for at least 200 days after inoculation. *tg*a*20* mice infected at *high dose* (n = 4 mice) or *low dose* (n = 5 mice) with RML6 succumbed to prion disease at 55 ± 2 (mean ± s.d., *high dose*) and 95 ± 5 (*high dose*) days, respectively.

To investigate the possible presence of prions in POM1-treated COCS in more detail, we assessed the prion propagation activity of antibody-treated *tg*a*20* COCS homogenates using the real-time quaking induced conversion assay (RT-QuIC) [10]. The RT-QuIC allows sensitive detection of PrP^Sc^ based on prion-catalyzed cyclic amplification of misfolded PrP. Homogenates of COCS treated with scFvPOM1 or scFvPOM1+recPrP_23-230_ as well as COCS homogenates treated with NBH- and RML6 were spiked into the RT-QuIC reaction mixture and amplified for 70 hours. Neither scFvPOM1-treated nor (scFvPOM1+recPrP_23-230_)-treated COCS homogenates induced *de novo* PrP aggregate formation in the RT-QuIC assay. In contrast, RML6-treated COCS homogenates induced aggregation after an initial lag phase of about 24 hours (Fig 2A).

The failure to detect PrP^Sc^ suggests that exposure to toxic GD ligands does not induce PrP aggregation. However, the sensitivity of the assays employed is not very high. Moreover, GD ligands may conceivably induce PK-sensitive PrP aggregates which may still be infectious [11]. Finally, cell-based assays suffer from the differential susceptibility of individual cell lines to specific prion strains, which is unpredictable and may lead to false-negative results [9].

To overcome these limitations, we tested the transmissibility of pathology from antibody-treated COCS to PrP^C^-overexpressing *tg*a*20* mice that serve as sensitive indicators of prion infectivity [7, 12]. COCS treated with scFvPOM1 or scFvPOM1+recPrP_23-230_ (1%, 18 μg total brain homogenate in 30 μl) were inoculated intracerebrally (i.c.) into *tg*a*20* mice. In control experiments (performed non-synchronously with mice of the same genotype and age), *tg*a*20* mice were i.c. inoculated with RML6 at high dose (1%, 36 μg total brain homogenate, corresponding to 3 x 10^6^ median lethal dose [LD_50_] units and low dose (0.0001%, 3.6 pg total brain homogenate, corresponding to 3 x 10^2^ LD_50_ units). RML6-infected mice showed the characteristic clinical signs of terminal prion disease (e.g. progressive ataxia, rolling gait) at 55 ± 2 (high dose) and 95 ± 5 days (low dose, Fig 2B). *tg*a*20* mice inoculated with homogenates from scFvPOM1 or scFvPOM1+recPrP_23-230_ treated COCS, as well as non-inoculated control mice, did not develop any signs of disease within an observation period of 200 days (Fig 2B).

**Fig 3:**
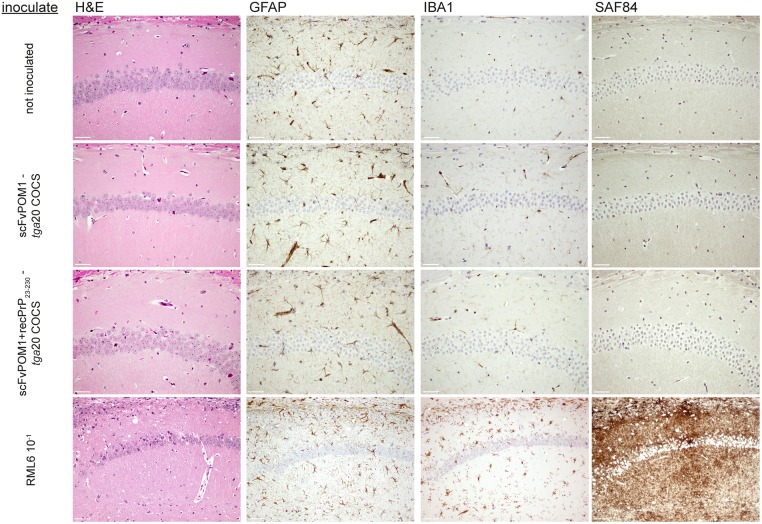
Brain sections from terminally sick mice (*high dose* RML6, lowest row) revealed typical signs of a prion disease such as spongiform change (H&E), astrogliosis (GFAP), microgliosis (IBA1) and PrP^Sc^ deposition (SAF84). None of these features were observed in *tg*a*20* mice inoculated with scFvPOM1-COCS, (scFvPOM1+recPrP_23-230_)-COCS, or in untreated mice. Scale bar = 50 μm.

**Fig 4:**
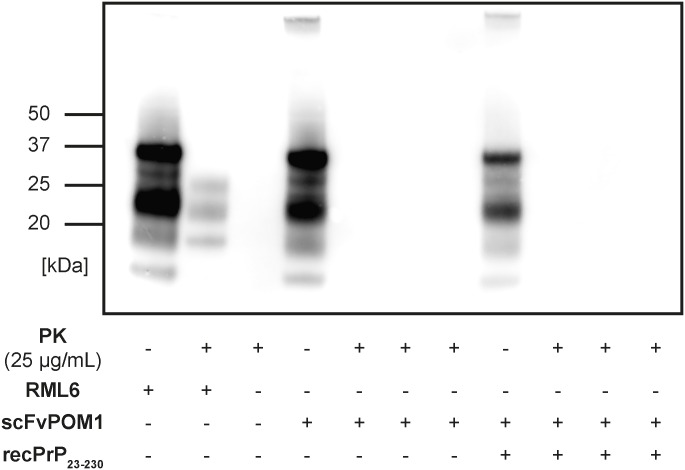
PK-digestion of brain homogenate from a terminally sick *tg*a*20* mouse revealed protease resistance. The residual PrP^Sc^ showed a typical diagnostic shift in its molecular weight. Neither untreated, scFvPOM1-COCS inoculated (n = 3 brain homogenates) nor (scFvPOM1+recPrP_23-230_)-COCS inoculated (n = 3 brain homogenates) *tg*a*20* mice showed detectable PrP^Sc^.

Immunohistochemical analysis of brain sections from RML6-inoculated mice showed prominent and classical signs of prion disease, such as spongiform changes, neuronal cell loss, astrogliosis, and microglial activation (Fig 3). In contrast, none of these signs were observed in mice treated with scFvPOM1 COCS homogenate, scFvPOM1+recPrP_23-230_ COCS homogenate, or non-inoculated mice (Fig 3). Western blots of brain homogenates from RML6-infected mice showed the molecular weight shift typical of PrP^Sc^ after PK digestion (Figs 4 and S3). No PrP^Sc^ was detected by Western blotting in brain homogenates from mice inoculated with scFvPOM1-COCS, (scFvPOM1+recPrP_23-230_)-COCS, or NBH.

## Discussion

The neurotoxic antibody POM1 mimics the typical prion pathology with spongiform change, neuronal loss, astrogliosis, and microglial activation, and triggers transcriptomic perturbations similar to those observed in prion diseases [4]. We have shown that prion toxicity or protection is dependent on the identity of the PrP^C^ epitopes recognized by each antibody [2]. Antibodies targeting the PrP^C^ globular domain are predominantly neurotoxic, whereas those targeting its flexible tail are largely protective [2]. FT-dependent protection through the OR-binding antibody POM2 is independent of prion replication, since it preserves neurons without reducing the accumulation of PK-resistant PrP^Sc^ [4]. PrP^C^ mutants devoid of the octapeptide repeat domain do not confer POM1 susceptibility to *Prnp*-deficient COCS, confirming that the FT is a crucial effector of neurotoxicity [2].

The scrapie prion protein PrP^Sc^ is defined through its resistance to enzymatic digestion by proteases and is commonly used as a surrogate marker for the presence of infectious prions [8], but it was unclear if, and to what extent, prion mimetics such as the monoclonal antibody POM1 were able to induce prions. We now report that scFvPOM1 treatment of COCS does not lead to generation of infectious prions. In view of the pivotal importance of this question, we addressed it with a set of orthogonal methodologies including a cell bioassay, real-time quaking induced conversion, and a sensitive bioassay utilizing PrP^C^-overexpressing transgenic mice.

The induction of toxicity independent of infectivity suggests the existence of a toxicity-inducing epitope of PrP^C^ that may be accessible by both neurotoxic antibodies and infectious prion seeds. The conformational POM1-epitope in the α1-α3 region of PrP^C^ could represent such a site, since additional monoclonal antibodies other than POM1 targeting residues in close proximity also induce neurotoxic effects [3].

Because of technological limitations, the docking of infectious PrP^Sc^ oligomers to membrane-resident normal PrP^C^ has never been observed directly. However, such docking represents a plausible basis for the phenomenon of prion toxicity. Accordingly, prions damage only neurons that express PrP^C^ on their surface, whereas Prnp-negative neurons do not experience degeneration even after chronic, long-term exposure to prions [13]. We therefore speculate that the docking of POM1 may mimic the process by which prions dock onto PrP^C^. Consequently, we predict that compounds conferring protection against POM1 may have a high likelihood to be protective against infectious prions–a prediction that has been verified experimentally with a number of compounds [4]. By extension, POM1 toxicity seems to represent a promising model of prion toxicity that might allow for screening prion-protective compounds without the need of advanced biosafety measures.

In conclusion, our data add to the growing body of evidence that prion protein-mediated toxicity is independent from prion infectivity, and hence favor a model of prion toxicity in which toxic prion antibodies operate downstream of prion replication.

## Materials and Methods

### Animal experimentation, animal welfare and ethics statement

All animal experiments were conducted in strict accordance with the Rules and Regulations for the Protection of Animal Rights (Tierschutzgesetz and Tierschutzverordnung) of the Swiss Bundesamt für Lebensmittelsicherheit und Veterinärwesen BLV. Body weights were measured weekly. All animal protocols and experiments performed were specifically approved for this study by the responsible institutional animal care committee, namely the Animal Welfare Committee of the Canton of Zürich (permit numbers 41/2012, 90/2013 and ZH040/2015). All efforts were made to minimize animal discomfort and suffering.

### Animal survival study, inoculation of prions and scFvPOM1-treated brain tissue

Prion inoculations were performed under isofluorane anesthesia. Monitoring of mice was perfomed as described previously [14]. Herein, mice were monitored after prion inoculations every other day and actions were taken to minimize animal suffering and distress as is laid out in S1 Table. Diagnosis of scrapie was undertaken according to appropriate clinical criteria, namely ataxia, limb weakness, front leg paresis and rolling. CO_2_ inhalation was used to euthanize mice on the day of appearance of terminal clinical signs of scrapie (S1 Table). Brains obtained at necropsy were either snap-frozen for biochemical analysis or fixed in 4% formalin for histological assessment. The time elapsed from prion inoculation to the terminal stage of disease was defined as incubation time for the survival study. All brain and COCS homogenates were diluted in 0.32 M sucrose in demineralized water before inoculation. 3 months-old *tg*a*20* mice were inoculated in the right frontal lobe at 1% (36 μg protein in 30 μL, corresponding to 3 x 10^6^ LD_50_ units, *high dose*) and 0.0001% (3.6 ng protein per animal, corresponding to 3 x 10^2^ LD_50_ units, *low dose*) of RML6 homogenate, and 1% (18 μg protein in 30 μL) of COCS homogenate. The protein concentrations in brain homogenates used for inoculation are in agreement with previously reported protein concentrations of infectious and non-infectious brain homogenates [15]. No unexpected deaths were observed during the animal experiments reported.

### Cerebellar organotypic slice cultures (COCS)

*tg*a*20* mice [7] were used for both COCS and prion inoculations. Preparation of COCS was performed as already described elsewhere [6]. 350 μm thick COCS were prepared from 9–12 day old *tg*a*20* pups. Antibody exposure was performed after a recovery period of 10–14 days after slice preparation. For preparation of slice homogenates, COCS were washed twice with PBS and homogenized in 0.32 M sucrose solution in demineralized water.

### Antibodies, recombinant full-length prion protein (recPrP_23-230_), prion inoculations and pharmacological treatment of COCS

Single-chain variable fragment antibodies of the IgG antibody POM1 (scFvPOM1) were produced through purification of inclusion bodies from *E*. *coli* on a Ni-NTA column, as reported before [2]. Bacterial expression of mouse recPrP_23-230_ was described elsewhere [16, 17]. COCS were inoculated with 100 μg brain homogenate per 10 slices from terminally sick prion-infected (RML6) or NBH from CD1 mice, diluted in 2 mL physiological Grey’s balanced salt solution as described before [18]. Brain slices were incubated with infectious brain homogenates as free-floating sections for 1 h at 4°C, then washed and 5–10 slices were placed on a 6-well PTFE membrane insert. 10–14 days after slice preparation, antibodies were added with every medium change (three times a week). When antibody was given together with recPrP_23-230_, addition to slices was preceded by pre-incubation of antibody-recPrP mix on ice for at least 1 hour.

### Real-time quaking induced conversion assay

RT-QuIC assays of antibody-treated COCS were performed using recombinant hamster prion protein (HaPrP) as substrate for PrP^Sc^-catalyzed conversion as previously described [10]. The gene encoding recombinant hamster full-length (23–231) PrP was cloned into the pRSET-A expression vector (Invitrogen) via the NdeI and EcoRI restriction sites [17]. HaPrP was expressed in Rosetta2(DE3)pLysS *E*.*coli* competent cells in Overnight Express Instant TB Medium (Merck Novagen) for 26 hours at 30°C and 90 rpm. Briefly, cells were harvested by centrifugation at 17 000 *g* for 5 min and lysed by repeated freeze-thaw cycles. Cytoplasmic inclusion bodies were then washed twice with each 1X and 0.1X BugBuster Protein Extraction Reagent (Novagen) and solubilized in 8 M GdnHCl (pH 8.0). After another centrifugation step at 17 000 *g* for 10 min, the supernatant was subjected to an affinity chromatography using Ni^2+^-nitrilotriacetic acid Superflow resin (QIAGEN). The protein was then refolded and purified on column by running a linear gradient from 100 mM NaH_2_PO_4_, 10 mM Tris-HCl, 6 M GdnHCl (pH 8.0) to 100 mM NaH_2_PO_4_, 10 mM Tris-HCl (pH 8.0) over 3.5 hours. After elution with a linear gradient to pre-cooled 500 mM imidazol, 100 mM NaH_2_PO_4_, 10 mM Tris-HCl (pH 6.0), the protein containing fractions were combined and filtered through a 0.2 μm syringe filter. The purified protein was dialyzed against 200 mM NaH_2_PO_4_ (pH 5.8) and concentrated to a final protein concentration of 0.6–0.8 mg mL^-1^ prior to storage at -80°C.

For the RT-QuIC assay, recombinant HaPrP was filtered using 100 kDa centrifugal filters (Pall Nanosep OD100C34) and adjusted to a final concentration of 0.1 mg mL^-1^ in PBS (pH 7.4) containing 170 mM NaCl, 10 μM EDTA, 10 μM Thioflavin T. RT-QuIC reactions were seeded with 2 μL scFvPOM1- or (scFvPOM1+recPrP_23-230_)-treated *tg*a*20* COCS homogenates (0.5 ng μL^-1^ total protein concentration in N2/PBS) in a total reaction volume of 100 μL. NBH- and RML6-treated *tg*a*20* COCS homogenates were used as negative and positive controls, respectively. All reactions were performed in quadruplicates in black 96-well polystyrene microplates (Nunc 265301) covered with sealing tape (Nunc 232702). The RT-QuIC reactions were amplified over a time period of 70 hours at 42°C with alternating cycles of 90 s shaking at 900 rpm in double orbital mode and 30 s resting using a FLUOstar Omega microplate reader (BMG Labtech). The amplification of *de novo* aggregates was followed real-time by measuring the increase in Thioflavin T fluorescence every 15 min (450/480 nm ex/em filters; bottom read mode).

### *In vitro* bioassay

*In vitro* assessment of prion propagation was performed with subclones of the murine neuroblastoma cell line CAD5 as already described [19]. Herein, the cells were exposed to 0.001% RML6 (12 ng in 1 mL), 0.001% non-infectious brain homogenate (NBH, 11 ng in 1 mL) and 0.001% scFvPOM1- or scFvPOM1+recPrP_23-230_ COCS homogenate (6 ng in 1 mL). Prions were allowed to propagate for 3 days followed by a 1:7 split, with three further 3-day growth periods and 1:7 splits. Cells were harvested, lysed in PBS with 1% Triton-X 100, Complete^™^ Protease Inhibitor and phosSTOP^™^ Phosphatase Inhibitor (Roche).

### Immunohistochemical stainings and NeuN morphometry

For immunohistochemical stainings, COCS were rinsed twice in PBS and fixed in 4% paraformaldehyde for 2 days at 4°C. COCS were then washed twice in PBS and incubated in blocking buffer (0.05% Triton X-100 vol/vol, 0.3% goat serum vol/vol in PBS) for 1 hour at room temperature. Alexa-488 conjugated mouse anti-NeuN antibody (Life Technologies) was diluted at 1.6 μg mL^-1^ in blocking buffer and incubated for 3 days at 4°C. Slices were washed for 15 min in PBS followed by a 30 min incubation with DAPI (1 μg mL^-1^) in PBS at room temperature. Slices were subsequently washed twice in PBS for 15 minutes and then mounted with fluorescent mounting medium (DAKO) on a glass slide. NeuN morphometry of COCS was undertaken on images taken on a fluorescent microscope (BX-61, Olympus) at identical exposure times through custom written scripts for the image analysis software cell^P (Olympus) as already described [2]. For immunohistochemistry of inoculated brains, formalin-fixed tissues were processed as described [16]. In brief, antibodies for detection of astrocytes (anti-GFAP; 1:300; DAKO) and microglia (anti-Iba-1; 1:1,000; Wako) as well as for detection of partially PK-resistant prion protein deposition (anti-PrP SAF-84; 1:200; SPI bio) where applied after the respective pre-treatment. Sections were developed with IVIEW DAB Detection kit (Ventana) and were then counterstained with hematoxylin as described [16]. Afterwards, sections were screened microscopically (Zeiss Axioskop 2 plus) for astrogliosis, microglia activation and prion protein deposition by two neuropathologists. Representative images were acquired using a digital camera (Olympus UC30).

### Protein analysis

Slices in cell culture inserts were washed twice in PBS and further scraped off the membrane with PBS (10 μL per slice), followed by trituration with a 30G syringe. Protein concentrations were measured by a bicinchoninic acid-based assay (Pierce^™^ BCA Protein Assay Kit, Thermo Fisher). For determination of PrP^Sc^, cell homogenates were digested with 5 μg mL^-1^, *tg*a*20* COCS and C57BL/6 whole brain homogenates were digested with 25 μg mL^-1^ PK (Roche) at a final volume of 20 μL in PBS for 30 minutes at 37°C. *tg*a*20* whole brain homogenates were digested with 50 μg mL^-1^ PK at a final volume of 20 μL in PBS for 45 minutes at 37°C. Loading buffer was added and samples were boiled at 95°C for 5 minutes to deactivate PK. Equal sample volumes were loaded on Nu-PAGE Bis/Tris precast gels (Life Technologies) and Western blotting was performed using the monoclonal anti-PrP antibody POM1 as described elsewhere [4].

### Statistical analyses

Unless mentioned otherwise, unpaired, two-tailed student’s T-test was used for comparing data from two groups and one-way ANOVA with Dunnett’s post-hoc test for comparing multiple groups. All data is given as mean ± standard deviation. Statistical analysis and data visualization was done using GraphPad Prism 6 (GraphPad).

## Supporting Information

S1 Fig Uncropped and unmodified Western blot of fig 1B with size markers. (TIF)

S2 Fig Uncropped and unmodified Western blot of fig 1C with size markers. (TIF)

S3 Fig Uncropped and unmodified Western blot of fig 4 with size markers. (TIF)

S1 Table Clinical assessment and scoring of *tga*20 mice inoculated with RML6 according to [14]. (DOCX)
