# Supplementary figures and images for: Neurotoxic Antibodies against the Prion Protein Do Not Trigger Prion Replication
Source: PLoS One. 2016 Sep 29;11(9):e0163601. doi: 10.1371/journal.pone.0163601 (PMC5042507; doi:10.1371/journal.pone.0163601)

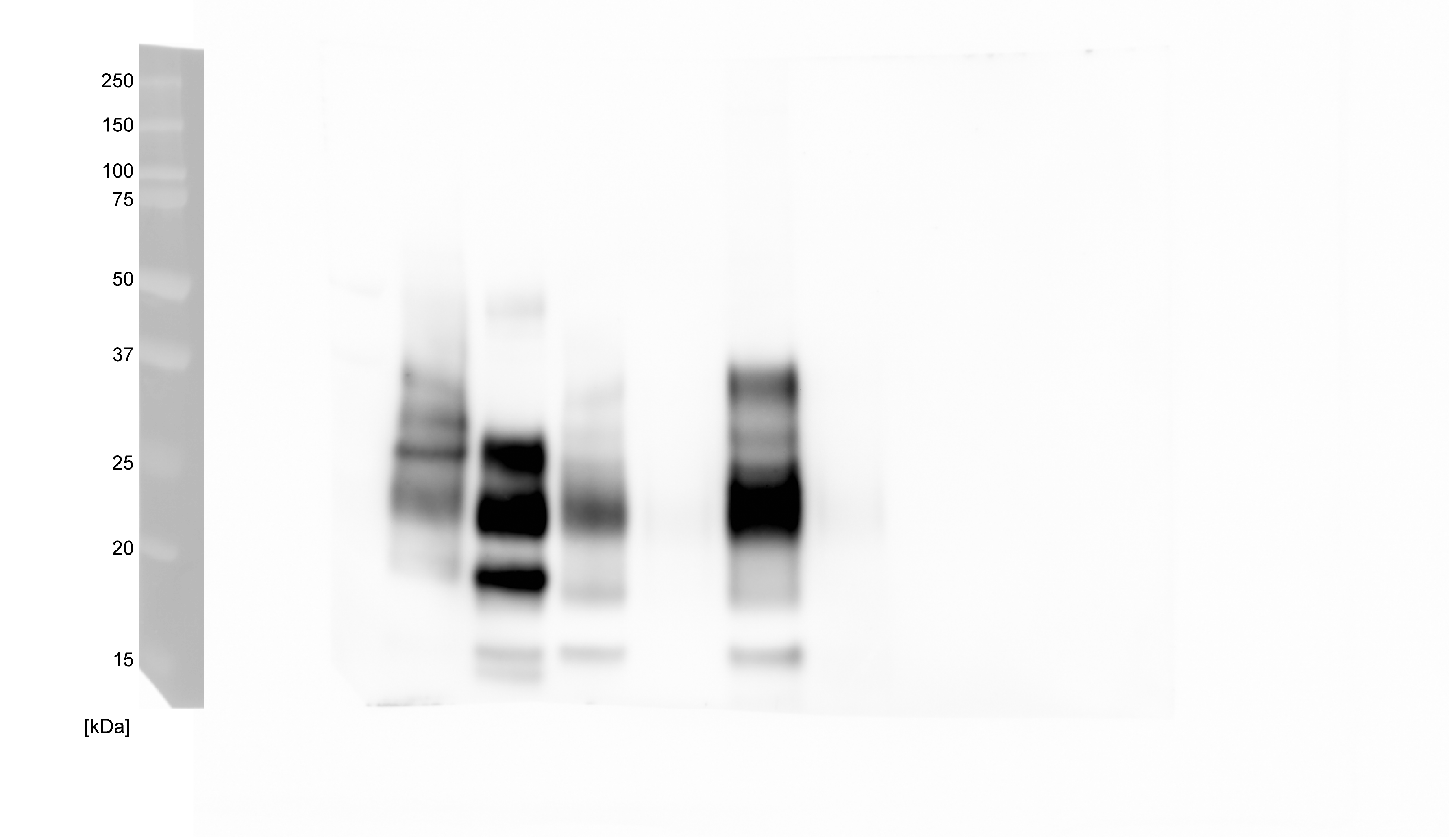

Supplement: S1 Fig — (TIF) [file pone.0163601.s001.tif]

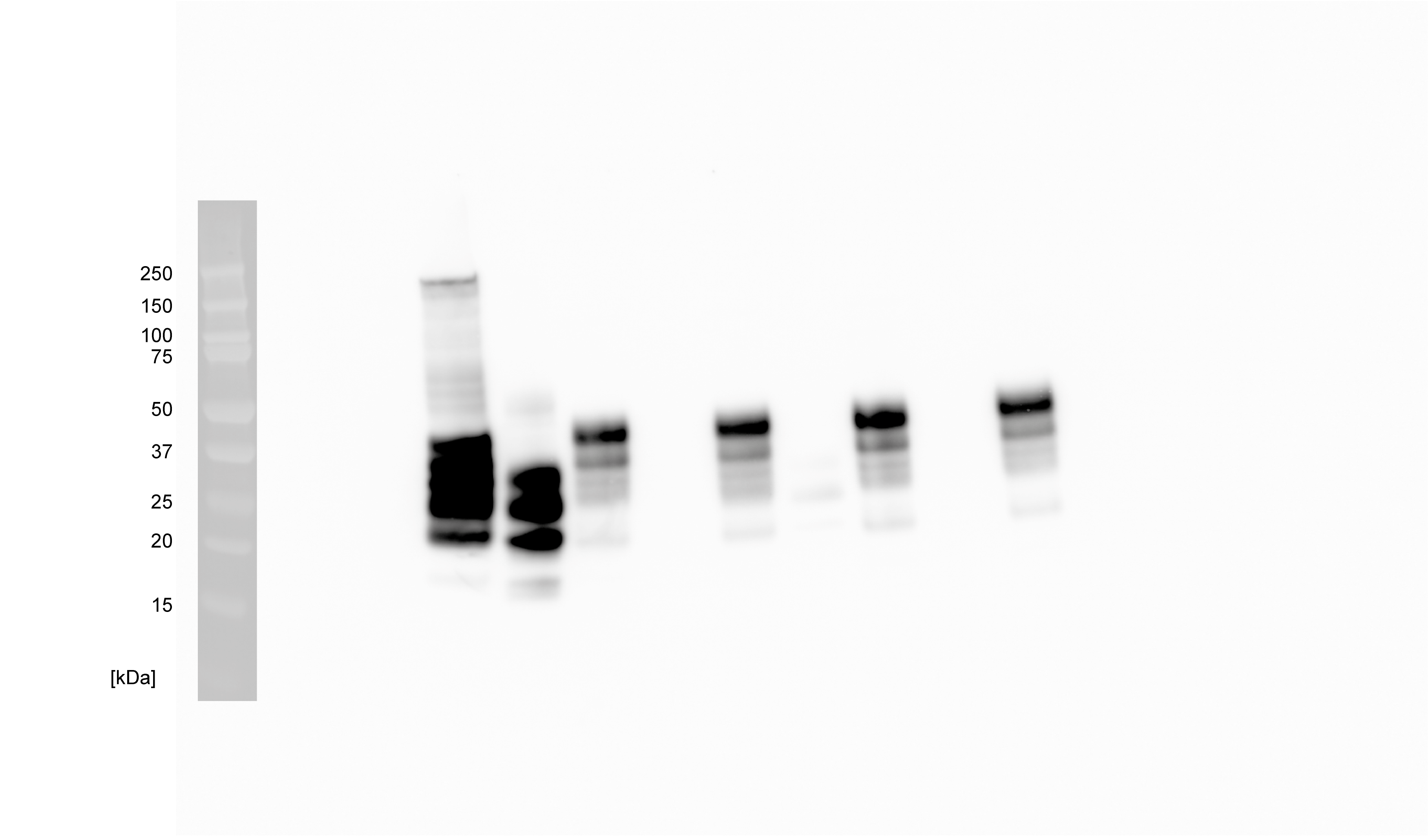

Supplement: S2 Fig — (TIF) [file pone.0163601.s002.tif]

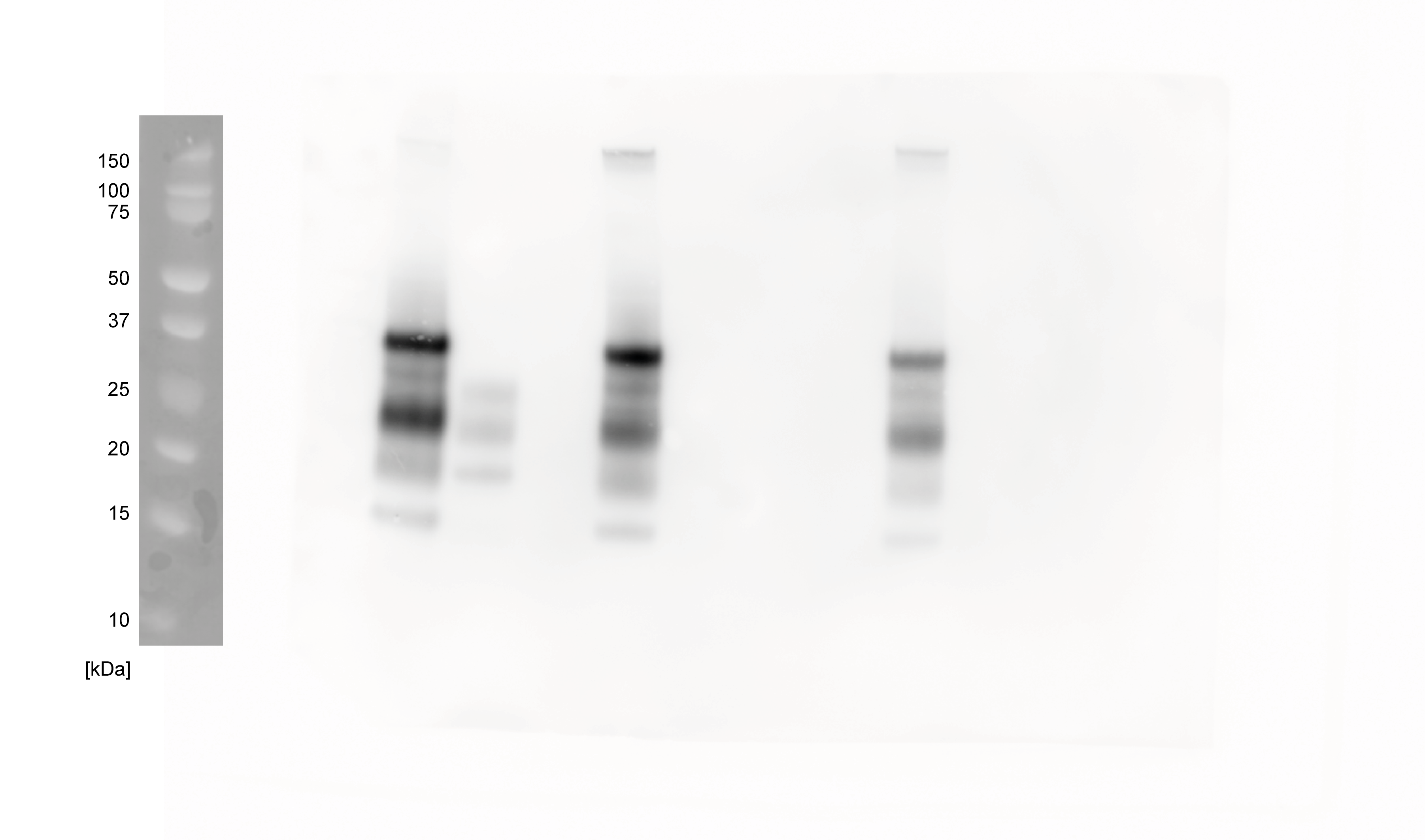

Supplement: S3 Fig — (TIF) [file pone.0163601.s003.tif]
