# Supplementary material for: Neurotoxic Antibodies against the Prion Protein Do Not Trigger Prion Replication
Source: PLoS One. 2016 Sep 29;11(9):e0163601. doi: 10.1371/journal.pone.0163601 (PMC5042507; doi:10.1371/journal.pone.0163601)
Supplement: S1 Table — (DOCX) [file pone.0163601.s004.docx]

**S1 Table: Clinical assessment and scoring of tga20 mice inoculated with RML6 according to [14]**

The animals were observed every other day after RML6 inoculation for clinical signs including gait, grooming, activity, rough hair coat, limb paresis and ataxia. Once the mice showed the first sign of scrapie (grade 1), they were monitored every day and wet food was supplied in the cage. When the mice reached score grade 2 that hindered them reaching the water bottle, they were euthanized by CO_2_ inhalation.

| **Score** | **Clinical signs** | **Assessment** | **Action** |
| --- | --- | --- | --- |
| 0 | No detectable signs of abnormal movement |  |  |
| 1 | Waddling gait, mild signs of reduced grooming, rough hair coat, limb weakness, front leg paresis | Slight rolling while shaking the cage | Provide wet food in the cage;  Observe every day |
| 2 | Ataxia, reduced grooming and activity, paralysis, rolling | Rolling while shaking the cage | Euthanize immediately once the clinical signs hamper the mice reaching the water bottle |
| 3 | Dead |  |  |
